# Assessing the effects of long-term osteoporosis treatment by using conventional spine radiographs: results from a pilot study in a sub-cohort of a large randomized controlled trial

**DOI:** 10.1007/s00256-018-3118-y

**Published:** 2018-12-01

**Authors:** Hans Peter Dimai, Richard Ljuhar, Davul Ljuhar, Benjamin Norman, Stefan Nehrer, Andreas Kurth, Astrid Fahrleitner-Pammer

**Affiliations:** 1 Department of Internal Medicine, Division of Endocrinology & Diabetology, Medical University of Graz, Auenbruggerpl. 15, 8036 Graz, Austria; 2 Image Biopsy Lab, Research & Development, Vienna, Austria; 3 Braincon Technologies, Vienna, Austria; 4 Department for Health Sciences and Biomedicine, Center for Regenerative Medicine and Orthopedics, Danube University Krems, Krems, Austria; 5 Klinikum Birkenwerder, Birkenwerder, Germany

**Keywords:** Osteoporosis, Long-term treatment, Bone structure value (BSV), Conventional radiograph, Dual X-ray absorptiometry (DXA)

## Abstract

**Objective:**

To evaluate the clinical applicability of a software tool developed to extract bone textural information from conventional lumbar spine radiographs, and to test it in a subset of postmenopausal women treated for osteoporosis with the fully human monoclonal antibody denosumab.

**Methods:**

The software was developed based on the principles of a fractal model using pixel grey-level variations together with a specific machine-learning algorithm. The obtained dimensionless parameter, termed bone structure value (BSV), was then tested and compared to bone mineral density (BMD) in a sub-cohort of postmenopausal women with osteoporosis who were treated with the monoclonal antibody denosumab, within the framework of a large randomized controlled trial and its open-label extension phase.

**Results:**

After 3 years and after 8 years of treatment with denosumab, mean lumbar spine BMD as well as mean lumbar BSV were significantly higher compared to study entry (one-way repeated measures ANOVA for DXA: F = 108.2, *p* < 0.00001; and for BSV: F = 84.3, *p* < 0.00001). The overall increase in DXA-derived lumbar spine BMD at year 8 was + 42% (mean ± SD; 0.725 ± 0.038 g/cm^2^ to 1.031 ± 0.092 g/cm^2^; *p* < 0.0001), and the overall increase of BSV was 255% (mean ± SD; 0.076 ± 0.022 to 0.270 ± 0.09, *p* < 0.0001). Overall, BMD and BSV were significantly correlated (*R* = 0.51; *p* < 0.0001).

**Conclusions:**

This pilot study provides evidence that lumbar spine BSV as obtained from conventional radiographs constitutes a useful means for the assessment of bone-specific treatment effects in postmenopausal women with osteoporosis.

## Introduction

Since the early 1990s, measurement of bone mineral density (BMD) by dual energy X-ray absorptiometry (DXA) has been the globally accepted “gold-standard” method for the noninvasive diagnosis of osteoporosis as defined by World Health Organization (WHO) criteria [1]. This is irrespective of the fact that the relevance of this imaging technology in daily clinical practice is limited by many factors, such as its low availability and its relatively high costs, which have to be considered in addition to conventional X-ray devices. Furthermore, both its sensitivity to discriminate individuals with osteoporotic fracture from those without, and its power to predict future fracture in individuals have been shown to be low [2]. In addition, longitudinal studies have shown that the rate of age-related bone loss of the spine, when measured by DXA in anterior-posterior projection according to the current recommendations of the ISCD (International Society for Clinical Densitometry) and IOF (International Osteoporosis Foundation), may not accurately reflect true changes, since aortic, sclerotic, and osteophytic calcifications may produce falsely elevated BMD values [3–5]. Notwithstanding, DXA has not only been widely accepted as the most important surrogate parameter and secondary endpoint in randomized controlled trials investigating the effect of bone active agents over time, but also as the most important indicator of treatment response during treatment monitoring in daily clinical practice [6, 7].

Against this background, efforts have been undertaken in the past decades to develop and establish imaging technologies (sometimes also referred to as imaging biomarkers) that would overcome the limitations of DXA. However, despite their clear superiority in regard to monitoring treatment effects and assessing bone strength, none of these advanced imaging devices such as magnetic resonance (MR), multi-detector computed tomography (CT), and high-resolution peripheral quantitative CT (HR-pQCT) has the potential of being integrated into daily clinical practice. This is primarily because of their high cost and their non-availability to the vast majority of health-care facilities. Even Trabecular Bone Score (TBS), a recently developed software tool that can be incorporated into existing DXA devices, and which has been shown to provide information in addition to that provided by BMD alone, cannot overcome the limitations caused by DXA itself, particularly in regard to its relatively low resolution and availability in daily clinical practice.

In contrast, conventional X-ray devices are available in almost any hospital worldwide in addition to devices available in radiology practices. Interestingly, and for reasons that are not really obvious, further development and investigation of information that could potentially be extracted from conventional radiographs has not been a matter of interest for many years, despite the fact that conventional X-ray imaging, particularly of the thoracic and the lumbar spine, still plays a key role in the basic evaluation of patients with suspected osteoporosis [8, 9]. In this regard, even in cases where vertebral fracture is suspected by using DXA-based vertebral fracture assessment (VFA), additional imaging with conventional radiography is recommended, particularly in cases where sclerotic or lytic changes, or findings suggestive of conditions other than osteoporosis are present [9].

Fractal-based analysis of trabecular bone has been shown to provide structural information by extracting three-dimensional information from two-dimensional plain radiographic images [10, 11]. Clinically, fractal-based analysis applied to trabecular bone of calcaneus radiographs has been shown to discriminate between patients with prevalent vertebral fractures from such without, and that the discriminative power of fractal analysis may even be superior to that provided by BMD measurement [11, 12]. In addition, it has been demonstrated that if applied to conventional radiographs of subchondral bone of the proximal tibia in patients with osteoarthritis, fractal-based analysis may provide reliable information on osteoarthritis progress, facilitating quantification of the severity and classification of this disease [13, 14]; and finally, it has been demonstrated recently that fractal-based image analysis, similar to the one used in the prevailing study, can be used to quantify the radiographic changes of subchondral bone in rheumatoid arthritis hands [15].

The aim of the present pilot study is to clinically asses the effects of treatment on osteoporosis using recently developed, fractal-based software designed to extract bone textural information from conventional spine radiographs, and compare the obtained results with DXA-derived BMD. Our hypothesis is that conventional spine radiographs can be used to accurately and semiquantitatively assess treatment response of osteoporosis.

## Subjects and methods

A sub-cohort of postmenopausal women with osteoporosis who were treated with the monoclonal antibody denosumab within the framework of a large randomized controlled trial termed FREEDOM and its open-label extension phase, was investigated [16–18]. In order to support the clinical findings of this study in regard to bone structural indices like BSV, a supplementary ex vivo study in human vertebrae was performed using high-resolution quantitative computed tomography (hr-QCT).

### Subjects

The FREEDOM pivotal trial was an international, randomized, placebo-controlled trial in postmenopausal women with osteoporosis. Women aged 60–90 years with a lumbar spine or total hip BMD T-score less than − 2.5 at either site but not less than − 4.0 at both sites were eligible to enroll in this study. Subjects received placebo or denosumab 60 mg subcutaneously every 6 months for 36 months, with daily supplements of ≥ 1000 mg of calcium, and  ≥ 400 IU of vitamin D. Overall, data from 7808 women were available in the FREEDOM trial, including 3902 in the denosumab group and 3906 in the placebo group. Denosumab is a fully human monoclonal antibody to the receptor activator of nuclear factor-κB ligand (RANKL), which blocks its binding to RANK, inhibiting the development and activity of osteoclasts, followed by suppression of bone resorption. Details of the study and the main results have been reported previously [16–18].

All women who completed the FREEDOM study (i.e., completed their 3-year visit) in either the denosumab or placebo arm were eligible to enter the 7-year open-label Extension Phase of the trial, provided all inclusion criteria were met [17, 18]. In the Extension Phase, all participants were scheduled to receive 60 mg denosumab subcutaneously every 6 months (± 1 month) with daily calcium and vitamin D supplementation. Results from 5, 6, and 8 years of denosumab exposure in women included in the extension trial have been published previously [17–19].

For the purpose of the study presented here, postmenopausal women who had been recruited at the Medical University of Graz (one out of 178 participating study centers) and who completed the 3-year FREEDOM study and the 5-year open-label extension were included in the analyses. The study was registered in the European Union Drug Regulating Authorities Clinical Trials (EudraCT) database (2007–001041-17), and approved by the ethics committee of the participating institution (EK-07-146-0807).

### BMD measurement

BMD at the lumbar spine was assessed by DXA measurement (HOLOGIC QDR 4500; HOLOGIC Inc., Bedford, MA, USA) according to the FREEDOM protocol [16]. Results were expressed in grams per square centimeter. Measurements were performed at the pivotal trial screening visit (year 0) and at year 3 (which was corresponding to the screening visit of the extension phase), and at years 1, 2, 3, and 5 (which correspond to years 4, 5, 6, and 8 of the entire study) of the extension phase (Fig. 2a and b) [17–19]. As the Medical University of Graz did not participate in the DXA substudy of the FREEDOM pivotal trial, lumbar spine DXA measurements were not available for years 1 and 2 of the pivotal trial. Furthermore, data from year 7 of the extension phase were not included in the analyses, as official results from this study phase had not yet been published as a peer-reviewed article.

Furthermore, for analyses as conducted in the present study, local read data have been used instead of central data.

### X-ray imaging

According to the FREEDOM pivotal trial study protocol, radiographs of the thoracic and lumbar spine were taken in anterior-posterior (a.p.) and lateral projection at baseline, years 2 and 3 [16]. During the Extension Phase, radiographs of the thoracic and lumbar spine were taken at baseline (which was corresponding to the end of the FREEDOM pivotal trial) and at years 2, 3, and 5 [17, 18]. Vertebrae were locally assessed, visually inspected, and excluded from analysis if fracture was present and height-loss was equal to or exceeded 20 % according to a semi-quantitative classification [20].

For BSV analysis, digitally stored radiographs were available in 16-bit DICOM format from which date of acquisition, the modality, and the pixel spacing were extracted. However, the current version of the analyzer used (IB LAB TX™ Analyzer, IBL, Vienna, Austria) only allows image processing in 12-bit depth, yielding a gray level range of 0 to 4096. Furthermore, due to the retrospective design of this pilot study and due to the fact that the underlying randomized controlled trial was carried out over a period of 8 years, standardized pixel size was not available. In fact, depending on the detector plates used, the following pixel sizes were available: 100, 111, 114, and 150 μm (Table 1).

**Table 1:** BMD (g/cm^2^), BSV and pixel size (PS; μm) for individual patients over the entire treatment period of 8 years. BMD and BSV values reflect the average value of at least two vertebrae eligible for evaluation

Patient #	Screening 1									Screening 2														
	Visit month 0			Visit month 12			Visit month 24			Visit month 36			Visit month 48			Visit month 60			Visit month 72			Visit month 96		
	0*/0**			1*/0**			2*/0**			3*/0**			4*/1**			5*/2**			6*/3**			8*/5**		
	DXA g/cm^2^	BSV	PS mm	DXA g/cm^2^***	BSV	PS mm	DXA g/cm^2^***	BSV	PS mm	DXA g/cm^2^	BSV	PS mm	DXA g/cm^2^	BSV#	PS mm#	DXA g/cm^2^	BSV	PS mm	DXA g/cm^2^	BSV	PS mm	DXA g/cm^2^	BSV	PS μm
1	0.695	0.050	111	n.a.	0.065	111	n.a.	0.080	111	0.939	0.123	150	0.941	n.a.	n.a.	0.916	0.115	110	0.959	0.206	150	1018	0.252	150
2	0.763	0.118	111	n.a.	0.075	111	n.a.	0.068	110	1025	0.089	111	1014	n.a.	n.a.	1051	0.096	110	n.a.	n.a.	150	n.a.	n.a.	n.a.
3	0.647	0.067	111	n.a.	0.056	111	n.a.	0.051	111	0.819	0.082	110	0.877	n.a.	n.a.	0.866	0.135	150	0.867	0.228	150	0.925	0.283	110
4	0.754	0.097	111	n.a.	0.065	111	n.a.	0.084	110	0.945	0.168	111	0.939	n.a.	n.a.	0.965	0.154	110	0.973	0.396	114	0.983	0.384	110
5	0.686	0.095	111	n.a.	0.080	111	n.a.	0.062	110	0.920	0.118	110	0.913	n.a.	n.a.	0.929	0.161	150	0.942	0.324	150	0.957	0.222	114
6	0.724	n.a.	n.a.	n.a.	0.052	111	n.a.	0.061	111	0.888	0.113	150	0.942	n.a.	n.a.	0.879	0.164	110	0.952	0.316	150	0.931	0.396	110
7	n.a.	0.071	111	n.a.	0.079	111	n.a.	0.050	111	0.948	0.058	110	0.955	n.a.	n.a.	1015	0.221	110	n.a.	n.a.	n.a.	n.a.	n.a.	n.a.
8	0.664	0.089	111	n.a.	0.095	111	n.a.	0.065	111	0.888	0.118	110	0.867	n.a.	n.a.	0.888	0.161	111	0.905	0.285	150	1019	0.301	110
9	0.710	0.118	111	n.a.	0.067	111	n.a.	n.a.	n.a.	0.859	0.202	110	0.801	n.a.	n.a.	n.a.	n.a.	n.a.	n.a.	n.a.	n.a.	n.a.	0.184	110
10	0.757	0.075	111	n.a.	0.052	111	n.a.	0.139	110	0.958	0.103	110	n.a.	n.a.	n.a.	n.a.	n.a.	n.a.	n.a.	0.230	114	n.a.	n.a.	n.a.
11	n.a.	n.a.	n.a.	n.a.	0.054	111	n.a.	0.087	111	n.a.	n.a.	111	n.a.	n.a.	n.a.	n.a.	n.a.	n.a.	n.a.	n.a.	n.a.	n.a.	n.a.	n.a.
12	0.745	0.079	111	n.a.	0.079	111	n.a.	0.161	111	0.979	0.120	111	0.983	n.a.	n.a.	1008	0.147	150	1023	0.370	114	1046	0.361	114
13	0.758	0.055	111	n.a.	0.061	111	n.a.	0.096	111	1039	0.097	110	1014	n.a.	n.a.	1046	0.136	111	1080	0.268	110	1151	0.212	150
14	0.751	0.054	111	n.a.	0.054	111	n.a.	0.036	111	0.937	0.076	111	0.996	n.a.	n.a.	1016	0.175	111	1062	0.224	110	1123	0.200	110
15	0.763	0.061	111	n.a.	0.096	111	n.a.	0.068	111	0.771	0.166	111	n.a.	n.a.	n.a.	n.a.	n.a.	n.a.	n.a.	n.a.	n.a.	n.a.	0.109	150
16	0.779	n.a.	n.a.	n.a.	0.088	111	n.a.	0.131	111	0.804	0.148	111	n.a.	n.a.	n.a.	n.a.	n.a.	n.a.	n.a.	n.a.	n.a.	n.a.	n.a.	n.a.
17	0.714	0.056	111	n.a.	0.160	111	n.a.	0.139	111	0.931	0.140	110	0.948	n.a.	n.a.	0.959	0.144	150	1009	n.a.	n.a.	0.986	n.a.	n.a.
18	0.720	0.056	111	n.a.	0.078	111	n.a.	0.110	111	0.989	0.145	110	0.963	n.a.	n.a.	1017	0.131	111	1098	0.141	150	1203	0.228	150
19	0.698	0.072	111	n.a.	0.059	111	n.a.	0.124	111	0.836	0.141	150	0.855	n.a.	n.a.	0.852	0.211	110	0.832	0.346	111		0.380	150
MEAN	0.725	0.076	111	n.a.	0.074	111	n.a.	0.090	110.8	0.915	0.123	116.7	0.934	n.a.	n.a.	0.958	0.154	121.7	0.975	0.278	132.5	1031	0.270	126.0
SD	0.038	0.022	0	n.a.	0.025	0	n.a.	0.036	0.43	0.075	0.036	14.82	0.061	n.a.	n.a.	0.069	0.034	18.57	0.083	0.075	19.67	0.092	0.090	19.79

*n.a.* not available

* FREEDOM pivotal trial;* n* = years

** Extension trial;* n* = years

*** According to study protocol, no DXA reports available

# = According to study protocol, no spine radiographs available

As evident from the table, pixel sizes of 150 μm were used only after year 3 of the pivotal trial, raising the possibility that the steep increase in BSV thereafter could be due to the larger pixel sizes used. However, correlation analysis did not show a significant association between BSV and pixel size (*R* ~ 0.170; *p* > 0.09).

### Defining the region of interest

**Fig. 1:**
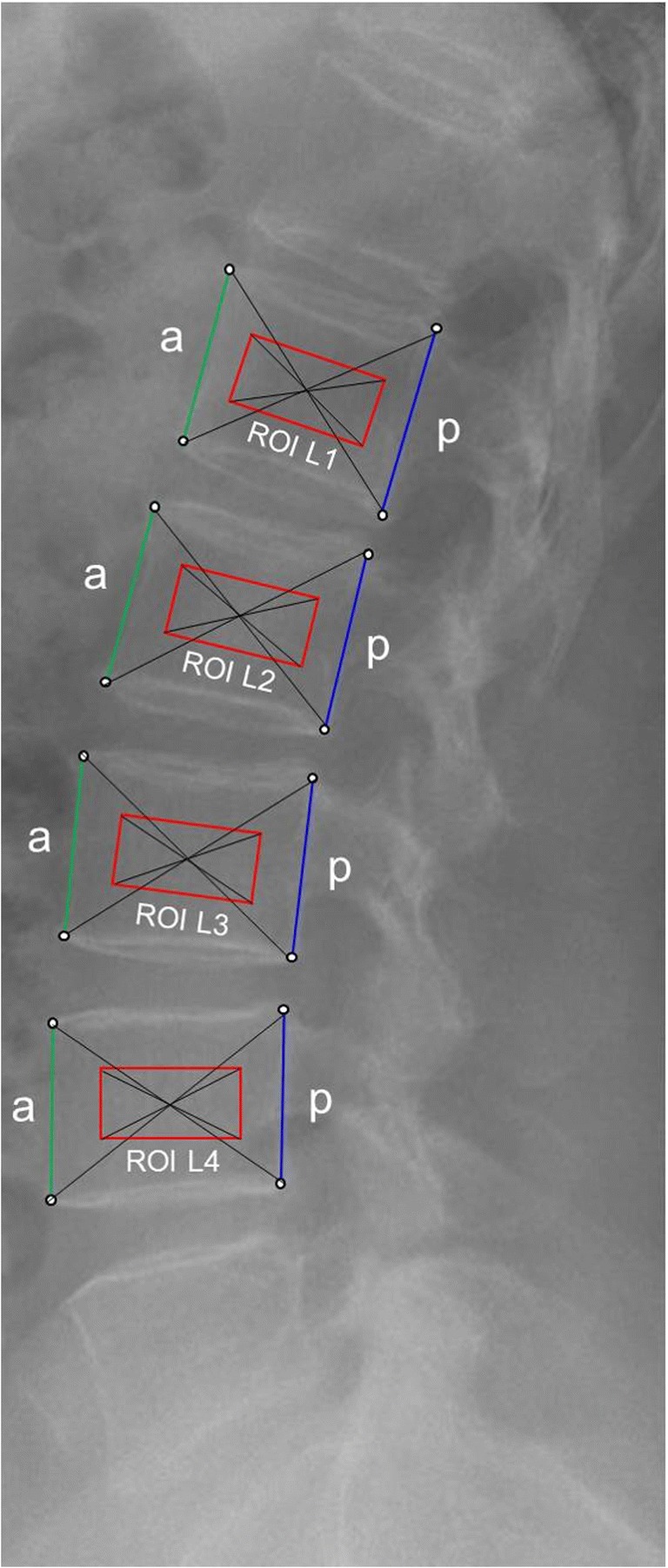
Semi-automatic ROI placement for measurement of BSV. Anterior (**a**) and posterior (**b**) edges of each vertebra were marked manually. ROIs sized 28 × 14 mm (*red rectangle*) were then set automatically so that the center of each vertebra was congruent with the center of the according ROI. The radiograph belongs to patient #3 (Table 1) and was taken at the screening visit in 2004

Digital radiograph images of the lumbar spine were analyzed using a semi-automatic software application (IB Lab TX Analyzer, IBL, Vienna, Austria). Thus, positioning of the ROI involved a two-step procedure as outlined in the following:

- In step one, anatomical landmarks were placed manually on the anterior and posterior edges of each of the vertebrae L1 – L4, depending on their respective eligibility (Fig. 1). If only three out of four vertebrae were eligible, then three vertebrae were analyzed, and if only two out of four were eligible, then only two were analyzed. If less than two of the vertebrae L1-L4 were eligible, the corresponding spine radiograph was excluded from further analysis.

Given that the landmarks on each vertebra constitute the technical pillars on which the consecutive, software-driven automated process is based, we sought to quantify potential intra- and interobserver bias. Thus, six different radiographs were studied by three observers (experienced radiologists), and each of these six radiographs was analyzed five times by each observer. The reading was done in a randomly selected order, with a minimum of 24 h between consecutive analyses. Observers were blinded to the respective results they had obtained previously. Both the anterior and the posterior height (mm) were measured for each vertebra, since (in a non-deformed vertebra) the crossing point of the diagonals between, e.g., the upper-posterior and the lower-anterior landmark is mainly determined by these parameters. For each series of five analyses of the same radiograph, a coefficient of variation (CV) was calculated and the results were then averaged, providing the final CV. The mean CV (%) for the intraobserver reproducibility was 0.60 ± 0.16 (SD).

The interobserver reproducibility was assessed by analyses performed by three observers (experienced radiologists) who were not identical with the observers of the intraobserver substudy. A total of six radiographs were analyzed by each radiologist, and the same parameters measured as for the intraobserver substudy. Observers were blinded to the results obtained by the other observers. A coefficient of variation (CV) was calculated and the results were then averaged, providing the final CV. The mean CV (%) for the interobserver reproducibility was 0.68 ± 0.4 (SD).

b) In step two, based on the manually placed landmarks, the final ROIs were then set automatically so that the center of each vertebra was congruent with the center of the according ROI (Fig. 1). The shape of the ROI was chosen as rectangular and their size was arbitrarily set at 28 × 14 mm, yielding a total of 2613 to 3920 pixels per ROI, depending on the detector plate used. Given that digitally stored radiographs were processed in 12-bit pixel depth, 4096 Gy-levels were available for analysis per ROI. For each ROI, the BSV was then calculated automatically, and the average BSV of all measurable vertebrae (i.e., at least two) was a patient’s final BSV result. However, in order to evaluate if ROI size would affect the BSV results, together with the chosen ROI of 28 × 14 mm, two additional ROI sizes were tested in each of the vertebrae L1-L4, in three randomly selected patients. The largest ROI was set manually so that the largest possible area of trabecular bone in each vertebra was captured, without including the end plates. The smallest ROI was set at 14 × 7 mm using the same semi-automatic software application as introduced above. The three different ROI showed no significant difference in BSV, but a highly significant correlation was observed (pairwise Mann–Whitney–Wilcoxon rank sum test: *p* > 0.4; one-way ANOVA: *p* > 0.85; multiple correlation coefficient: 0.99, *p* < 0.0001).

### Derivation and calculation of the BSV

The BSV, as performed in the prevailing study, is based on the principle of a fractal model, which has been described in more detail previously [21]. In short, fractals are a class of mathematical functions which can be used to characterize the geometrical properties of sets [21]. Accordingly, fractals can be applied to relate a metric property such as the length of a line (two-dimensional) or the area of a surface (three-dimensional) to the elemental length or area used as a basis for the calculation [21]. Two-dimensional patterns as present on plain radiographs of trabecular bone are thus well suited to fractal-based analyses. In fact, it has been shown that this two-dimensional information can be well translated into three-dimensional textural information by the use of a fractal model which involves the so-called fractional Gaussian noise, with the latter being the increment of the fractional Brownian motion (FBM) [22]. The only parameter of interest in the FBM is the so-called H parameter, also referred to as the Hurst exponent [21]. In order to assess this parameter, a maximum likelihood estimator must be applied [21]. This very method has been shown to yield high intra- and inter-observer as well as long-term reproducibility, with coefficients of variation being 0.61, 0.67, and 2.07%, respectively [22]. Based on the principles described above, oriented textural analysis software was developed, using grey-level variations together with a specific machine-learning-based algorithm. All vertical and horizontal pixel lines within each rectangular ROI (i.e., depending on the detector plates used: 187–280 vertical lines, and 93–140 horizontal lines, respectively) were analyzed in such way that each pixel (or gray-value) of a line was compared to each pixel (or gray-value) within the same line, yielding a vertical BSV (BSV_v_) as well as a horizontal BSV (BSV_h_). The final result for each ROI, and hence its textural characterization, was the BSV value obtained by averaging the BSV_v_ and BSV_h_ results of all lines within the same ROI, and the final result for the respective lumbar spine (or patient) was obtained by averaging the BSV value of all vertebrae included in the analysis. It should be noted that BSV is a unitless value.

### Supplementary ex vivo study

In order to test the hypothesis that BSV, as utilized for the clinical pilot study presented herein, provides information on structural indices of (trabecular) bone, we also carried out a supplementary ex vivo study. For this purpose, ten human vertebrae (five taken from a 67-year-old male patient, and five taken from an 87-year-old female patient; provided by the Macroscopic and Clinical Anatomy section of the Gottfried Schatz Research Center for Cell Signaling, Metabolism and Aging of the Medical University of Graz, Austria) was measured using two different technologies: high-resolution computed tomography (HR-pQCT; XTreme CT II, SCANCO Medical, Switzerland), and conventional X-ray (Multix Fusion Max, Siemens Healthcare, Germany).

## Statistical methods

A *p* value of ≤ 0.05 was set as the threshold for statistical significance. Descriptive statistics were used to summarize patient characteristics. Continuous variables were reported as means ± standard deviations. Changes in BMD and BSV over the treatment period of 8 years were analyzed by one-way repeated measures ANOVA. To assess the strength of a possible association between DXA-derived BMD and BSV, Pearson correlation coefficients were calculated. Statistical analyses were performed using SPSS Statistics software (IBM) and Python-based (version 2.7.0.) open-source software SciPy 0.18.1.

## Results

Between October 2004 and April 2005, a total of 32 postmenopausal women were recruited for the FREEDOM pivotal trial at the University Hospital of Graz, Austria, 23 in the denosumab group, and nine in the placebo group. Patients were not included in the present study if less than two consecutive BMD reports and/or spine radiographs were available. Patients randomized to the placebo group were not included in the analyses because the number of eligible subjects in the placebo group did not meet sufficient size to be included in the statistical analyses. Overall, of those patients randomized to the treatment group, 19 were eligible for inclusion into the present study. At study entry of the FREEDOM pivotal trial (Screening 1), mean age of the 19 treatment-group subjects included in the present analyses was 68.8 ± 5.4 years.

Over the entire study period of 8 years, the total number of lumbar spine radiographs available for BSV analyses was 110 (of 133 possible), and the total number of spine DXA reports available was 87 (of 114 possible) (Table 1).

At study entry of the FREEDOM pivotal trial, DXA-derived mean ± SD BMD at the lumbar spine was 0.725 ± 0.038 g/cm^2^, and mean ± SD BSV was 0.074 ± 0.022. After 3 years and after 8 years of treatment with denosumab, mean BMD as well as mean BSV were significantly different from their respective baseline values at study entry (DXA: F = 108.2, *p* < 0.00001; BSV: F = 84.3,* p* < 0.00001).

**Fig. 2:**
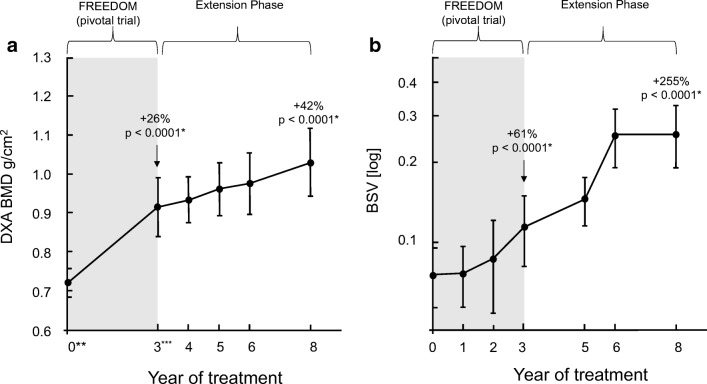
**a** Effect of denosumab 60 mg once subcutaneously every 6 months on BMD (g/cm^2^; mean ± SD). **b** Effect of denosumab 60 mg once subcutaneously every 6 months on BSV (mean ± SD)

Post hoc analysis revealed a steep and highly significant increase in BMD from baseline at study entry to year 3 (i.e., baseline of the extension phase; 0.725 ± 0.038 g/cm^2^ to 0.915 ± 0.075 g/cm^2^; *p* < 0.00001; +26%), and a significant increase from year 3 to year 8 of treatment with denosumab (1.031 ± 0.092 g/cm^2^; *p* < 0.001; +13%; Fig. 2a). The overall BMD increase from study entry to year 8 of treatment was + 42%.

A similar pattern, albeit more pronounced, was observed for BSV results, which showed a significant increase from baseline to year 3 of treatment with denosumab (0.076 ± 0.022 to 0.123 ± 0.036,* p* < 0.00; + 61%), and a steep and highly significant increase from year 3 to year 8 (0.270 ± 0.09, *p* < 0.0000; overall increase + 119%; Fig. 2b). The overall increase from study entry to year 8 of treatment was 255%.

**Fig. 3:**
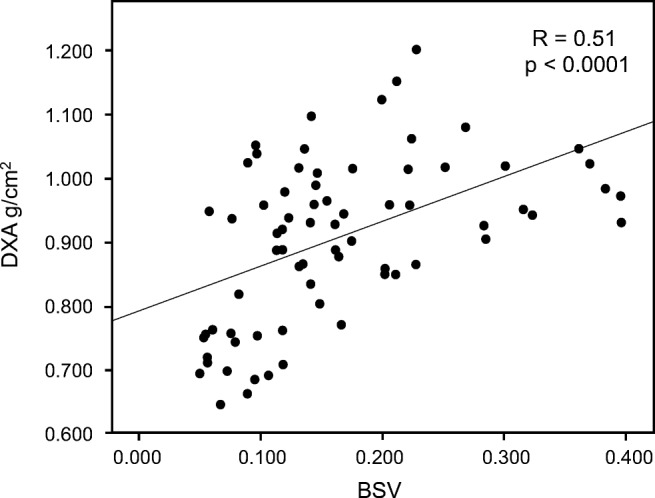
Correlation between DXA-derived BMD and fractal-based BSV

DXA-derived lumbar spine BMD and BSV showed a highly significant correlation (*R* = 0.51; *p* < 0.0001; Fig. 3).

### Supplemental HR-QCT study (ex vivo)

**Fig. 4:**
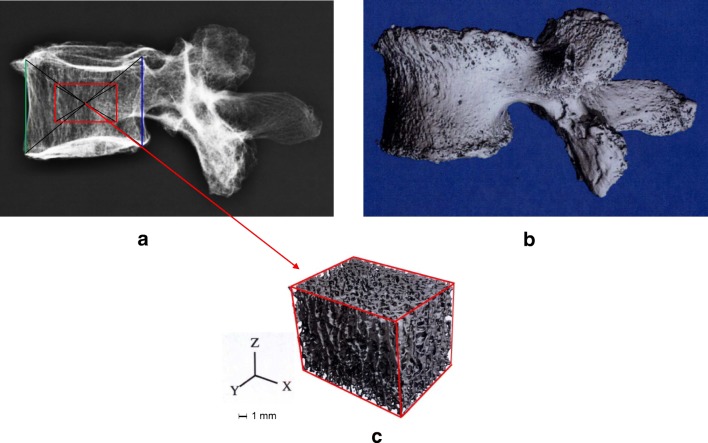
Panel showing ex vivo images of one and the same vertebra (lumbar vertebra 4 of an 87-year-old woman) taken with conventional radiograph (**a**), and high-resolution computed tomography (HR-pQCT; **b**). **c** showing the three-dimensional region of interest used for the assessment of histomorphometric parameters (e.g., trabecular bone volume)

Total volume (T.V.; mm^3^; *r* = 0.73, *p* < 0.05), bone volume (B.V.; mm^3^; *r* = 0.64,* p* < 0.05) and bone volume over total volume (BV/TV %; *r* = 0.52,* p* < 0.05) showed a significant correlation with BSV, providing direct evidence that in vivo assessment of vertebral BSV is an indicator of structural properties of vertebral trabecular bone (Fig. 4). However, trabecular number (Tb.N.;* r* = 0.5) and trabecular thickness (Tb.Th; *r* = 0.54) did not show a significant correlation with BSV.

## Discussion

The pilot study presented herein provides evidence that BSV as obtained from plain radiographs taken in lateral projection provides information on bone-specific treatment-related effects of denosumab. In the sub-cohort of postmenopausal women who had participated in the FREEDOM pivotal trial, BSV showed a marked and significant increase within the first 3 years after initiation of treatment, with a steep and sustaining increase during the extension phase up to 6 years of treatment, followed by a leveling-off between years 6 and 8. In more detail, the BSV increase at year 3 was as high as 61% compared to baseline, and the overall increase after 8 years of treatment was 255%. Although the pattern of increase in DXA-derived areal BMD (aBMD) was found to be similar and also well in line with increases published previously, it should be noted that the extent of increase in BSV was manifold higher compared to the one observed with aBMD [16, 18]. Currently, it can only be speculated as to why the response characteristic of BSV appears to be more “sensitive” towards a treatment with denosumab than DXA-derived aBMD. At least two possible reasons may be considered: First, BSV may not only capture (three-dimensional) textural properties of trabecular bone as shown previously and also in our supplementary HR-pQCT study, but also (two-dimensional) areal aspects such as bone mineral content. Second, it could be speculated that denosumab, independently of its effect on bone mineralization, would also result in some structural changes at vertebrae of the lumbar spine. In fact, structural changes in terms of an increase in cortical thickness and a decrease in cortical porosity in response to treatment with denosumab have been shown at least for the distal forearm, and it can therefore not be entirely precluded that such structural changes of the cortical bone of vertebrae may also have affected BSV results [23]. In addition, it cannot be precluded that treatment with denosumab may result in improvement of the vertebral trabecular structure, although such changes have not been shown before in vivo at the lumbar spine. This is simply due to the fact that hitherto neither invasive nor non-invasive adequate means have been available to provide direct evidence of such changes at the trabecular level. On the other hand, 3-year QCT data of the spine from a prespecified QCT sub-study of women from the FREEDOM trial showed a significant increase in (true) volumetric BMD (vBMD) in denosumab-treated subjects at 36 months by 22% [24]. This relatively large increase in vBMD might also have contributed to the size of the increase in BSV as observed in the present study.

It is of note that the increase in BSV in the present study showed a leveling-off after 6 years of treatment with denosumab. Again, at least two possible reasons might be considered: first, the further and sustaining increase in BMD with increasing study duration could at least in part be due to increasing degenerative changes such as osteophytes, which clearly have been shown to affect BMD results [4]. Second, histomorphometric data obtained from patients on long-term treatment with denosumab have recently shown that after 5 years of treatment, the mean degree of mineralization did not increase, and the heterogeneity index (HI) of the distribution of DMB did not decrease further, respectively [25]. BSV, being a parameter that has been shown to capture textural information, may thus provide even more reliable information on bone quality than DXA-derived BMD.

The study presented here showed a highly significant correlation between BSV and DXA-derived aBMD. This is not surprising, as BSV, aside from providing information on textural properties of trabecular bone, would also capture indices of mineralization, such as aBMD. The correlation between BSV and aBMD found in the present study was even more pronounced than the correlation between DXA-derived BMD and QCT-derived vBMD at the lumbar spine as recently described in postmenopausal women (*r* = 0.51 vs. 0.35) [26]. Furthermore, it is notable that in the same study the correlation between TBS, which has been shown to provide structural information, and vQCT of the lumbar spine was relatively weak and not different from the correlation between aBMD and vBMD. Unfortunately, no studies are available so far on the association between BSV and vBMD as measured at the lumbar spine.

In order to support the findings of the clinical pilot study presented here, a supplementary ex vivo study was carried out by measuring human vertebrae with both HR-pQCT and BSV. The results of this supplementary study provided strong supportive evidence that vertebral BSV as obtained from conventional radiographs is directly related to the (trabecular) bone volume of the vertebrae. Furthermore, intra- and interobserver reproducibility were tested in a smaller subset of radiographs taken from the present clinical pilot study, and the CVs obtained appeared to be reasonable in the context of a retrospective clinical study. Although the prevailing study was meant to be a pilot study, and although the cost for BSV measurement for routine clinical use have not yet been determined, it should be noted that BSV, as compared to possible other structural methods like MRT and HR-pQCT, may be considered as very cost-effective, since all it takes is a suitable X-ray device and a standard computer hardware to run the BSV software.

There are several limitations of this study that should be considered when interpreting the results. First, as already mentioned in the Materials and methods section, it should be kept in mind that assessment of BSV was carried out on radiographs that were taken more than a decade ago, implicating that image resolution was relatively low compared to resolutions provided by present-day X-ray devices and standards. Second, although radiographs had been taken according to recommendations outlined in the study protocol of the FREEDOM study, no standards have been given in regard to technical requirements, such as minimum resolution or voltages (kV) to be applied. Therefore, it cannot be entirely precluded that detector differences, particularly differences in pixel size, may have affected the study results. Third, the quality of the radiographs analyzed in this study not only depended on the X-ray devices (including their respective imaging detectors) used at that time, but also on the methodology used to digitally archive these images. On the other hand, given these limitations, it is even more noticeable that results obtained from BSV measurement in such radiographs appeared reasonable and well in accordance with the pattern obtained from the respective DXA measurements. In addition, it should be kept in mind that the prevailing study has been planned and designed only as a pilot study. Hence, one of its main purposes was to serve as a feasibility study, aiming to improve our understanding of the importance of image acquisition standards, and digital image storage standards, respectively, with the consequence of having clear standards available for a consecutive larger-scale trial.

In regard to the supplementary HR-QCT study performed within the framework of the prevailing pilot study, the number of post-mortem vertebrae investigated has been relatively low. Thus, there is a clear need for larger-sized studies in order to improve our understanding of the association between BSV and three-dimensional bone parameters.

Nevertheless, it is noticeable that despite the relatively low number of vertebrae investigated, a significant correlation was found between BSV and bone volume (BV), and the ratio of bone volume over total volume (BV/TV), respectively.

Finally, the relatively low number of subjects included in our study and the lack of a direct comparison with a placebo-group, together with a relatively high number of unavailable DXA results and/or radiographs, may limit the interpretation of the results. On the other hand, the FREEDOM sub-cohort analyzed in the present study constitutes a unique opportunity to assess changes in BMD and BSV under the umbrella of a large randomized controlled trial carried out over a period of 8 years. Furthermore, to the best of our knowledge, the supplemental ex vivo study presented herein is the first that provides direct evidence for a close association between trabecular indices of bone and BSV in human vertebrae.

In conclusion, this pilot study provides evidence that BSV as obtained from conventional spine radiographs taken in lateral projection constitutes a responsive means in the assessment of treatment-related effects in osteoporotic postmenopausal women treated with denosumab. In order to overcome these limitations, which have been caused primarily by the retrospective nature of the present study, prospective studies with clearly defined standards in regard to imaging acquisition need to be performed. In addition, further studies are required to identify the potential of BSV to be a measure not only of treatment effects but also of future fracture risk in untreated individuals with or without osteoporosis.
